# The Key Role of Amino Acids in Pollen Quality and Honey Bee Physiology—A Review

**DOI:** 10.3390/molecules29112605

**Published:** 2024-06-01

**Authors:** Maciej Sylwester Bryś, Aneta Strachecka

**Affiliations:** Department of Invertebrate Ecophysiology and Experimental Biology, University of Life Sciences in Lublin, Doświadczalna 50a, 20-280 Lublin, Poland; aneta.strachecka@up.lublin.pl

**Keywords:** mono diet, amino acid, metabolism, honey bee, nutrition, digestion

## Abstract

When studying honey bee nutrition, it is important to pay attention not only to the quantity but also to the quality of pollen for floral visitors. The recommended way to determine the value of pollen is to determine both the protein concentration and the amino acid composition in the insect’s hemolymph. In addition, the composition of pollen also includes lipids, sterols and biogenic elements such as carbon, nitrogen, etc. Very high protein concentration is observed in aloe pollen, averaging 51%. Plants with a high protein content, at the level of 27% in Europe, are rapeseed and phacelia. In turn, a plant that is poor in protein (at the level of 11%) is buckwheat. The aforementioned plants are sown over very large areas. Vast acreages in Central and Eastern Europe are occupied by pollen- and nectar-providing invasive plants, such as goldenrod. Therefore, bees are forced to use one food source—a mono diet—which results in their malnutrition. In the absence of natural pollen, beekeepers use other foods for bees; including soy protein, powdered milk, egg yolks, fish meal, etc. However, the colony is the strongest when bees are fed with pollen, as opposed to artificial protein diets. More research is needed on the relationship between bee pollen composition and nutrition, as measured by protein concentration and amino acid composition in apian hemolymph, colony strength, honey yield and good overwintering.

## 1. Introduction

**Figure 1:**
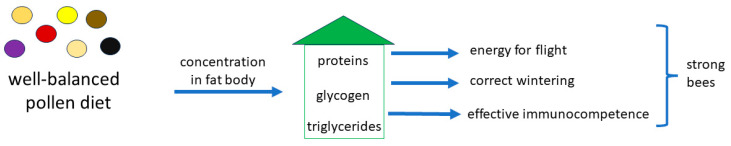
The effect of a well-balanced pollen diet on honey bee fat body and physiology of the honey bee.

The topic of pollinating insects’ diets has recently come to the forefront of scientific debate [1,2]. An improper diet disrupts the functioning of the gastrointestinal tract, leading to impaired absorption of nutrients; and promotes the development of pathogenic fungi, e.g., *Nosema* spp. [3]. Nutritional stress caused by a mono diet or lack of food results in premature mortality of bee colonies. The physiological nutritional needs of honey bees are not well understood, unlike other farm and companion animals [4]. Bees use flower resources to provide food. Nectar is the main source of simple sugars such as fructose and glucose [5]. In the process of glycogenesis, glucose is transformed into glycogen. Glycogen and triacylglycerols are stored in the cells of the fat body (trophocytes) [6,7]. Compounds such as glycogen, triglycerides and proteins are used to generate energy for ‘fuel’ during flight, for wintering purposes and for basic metabolic processes (Figure 1). A natural source of easily digestible protein, lipids, vitamins and minerals for bees is pollen [8]. A honey bee consumes it after lactic fermentation, in the form of bee bread [9]. The demand for bee bread depends on the apian genotype, the availability of floral resources around the apiary, the number of workers, the number of larvae and the accumulated resources [10]. An average honey bee colony collects from 15 to 55 kg of pollen per season [11]. The environment around the apiary plays a key role in the nutrition of honey bees, as it provides or does not provide protein food resources that vary throughout the year [4,12]. Protein-rich diets, including the pollen diet, determine a significant overlap in proteome expression patterns and influence the nutritional and metabolic effects in flower visitors [13]. In addition, a well-balanced diet determines the body’s homeostasis through the correct concentration of reserve compounds stored in the fat body. Currently, the relationship between the species diversity of pollen-producing plants and the metabolism of flower-visiting insects is still insufficiently understood [14]. Lack of availability and poor quality of nutrients contained in pollen affects some plant species, so it is important to ensure a variety of food. When talking about the diet of honey bees and other floral visitors, nutritional balance must be taken into account. Moreover, it is not enough to use random mixtures of bee-friendly plants, but they should be selected for their nutrient composition [15]. It is important to understand the foraging preferences of honey bees and other floral visitors throughout the season in different ecosystems. Beekeepers should take into account specific recommendations, such as limiting lawn mowing for the cultivation of *Taraxacum* sp., *Ranunculus* sp., etc., which will help to properly balance the bees’ diet [16]. Moreover, the above actions will promote the number of plant species and their numerical strength within the particular species, to increase the population of pollinators. However, these measures are still not sufficient and must evolve to consider what we have learned so far. An important scientific discovery was the identification of the segmental structure of the fat body in *Apis mellifera* L. [6,7]. The segmentation lends it a multi-tasking character, which makes the fat body comparable to the liver, spleen, pancreas and adipose tissue of vertebrates. Trophocytes and oenocytes that build the fat body participate in the energy metabolism of carbohydrates, lipids and proteins. Currently, there are many unanswered questions related to the pollen diet and its impact on the segmental structure of the fat body.

The aim of this review is a comprehensive analysis of the literature and a summary/collection of the current and most important data on the impact of amino acids contained in pollen on the physiology of honey bees.

## 2. Composition of Bee Pollen

**Figure 2:**
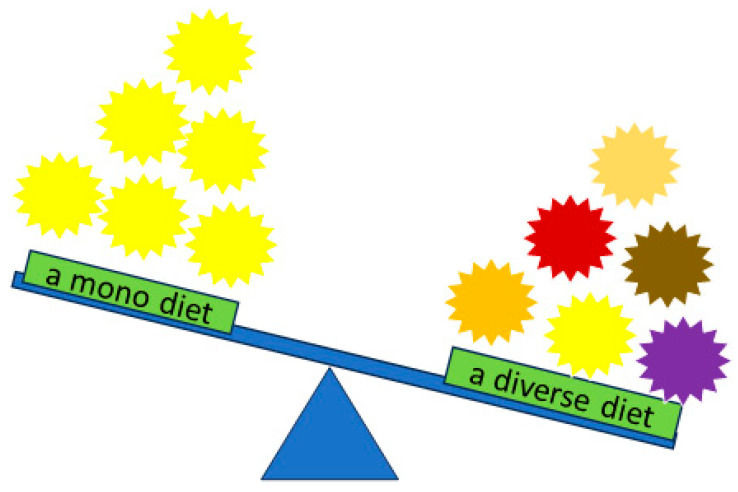
A diverse pollen diet contains different amino acids than a mono diet.

Scientists define plants as pollinator-friendly based on the amount of pollen or nectar produced, not its chemical quality [17]. Moreover, recommendations regarding plants that are valuable to insects, widely available to farmers, beekeepers and scientists, are usually inconsistent and unfocused [16]. When scientific data reports mention the biochemical composition of pollen, they usually refer to the crude protein content. This value ranges from 2.5 to 60% of the dry weight of angiosperm pollen [18,19,20]. A honey bee colony needs a diet containing approximately 20–25% protein for its proper growth and survival [19]. Amino acids and proteins present in the diet of bees should come from the pollen of various plant species (Figure 2). Therefore, it is widely acknowledged that a mono diet is not a balanced diet. Among the exceptions are plants such as *Castanea* sp. and *Trifolium* sp., which produce pollen with a high protein concentration (Table 1). We are not able to document all plant species for the physicochemical content of pollen. Scientists are focusing on the protein content of pollen from plants that dominate the diet of honey bees. Some plants, such as aloe (*Aloe Greatheadii* var. *davyana*), produce pollen with a high total protein content. There are also insect-pollinated plants in the environment that can produce pollen with a low protein content; e.g., sunflower (*Helinathus annus* L.). Freshly collected aloe pollen for worker bees is characterized by a high protein content (51%), as opposed to sunflower pollen (26%). Therefore, aloe pollen is preferred for overwintering bees by African beekeepers [21]. The pollen of *Echium plantagineum* L. [22] contains a high percentage of protein, at 37.4%. Buckwheat pollen (*Fagopyrum esculentum* Moench) is quite low in protein (11.4%). Buckwheat pollen, in turn, has a diverse composition of amino acids—especially exogenous amino acids—which enhances its nutritional value [22].

The percentage of protein in the insect diet changes with the season and, therefore, with the frequency of the flowering of pollen-bearing plants. In pollen samples collected from Mediterranean countries, the highest protein concentration was found in the spring pollen, while lower concentrations were found in samples from late summer [37,38]. Researchers have observed a similar trend of decreasing protein content with subsequent seasons in countries surrounded by the Atlantic Ocean and the region bordering the Pacific Ocean [19,39,40]. DeGrandi-Hoffman et al. [41] compared the protein content in pollen mixtures collected in pollen traps in spring and autumn. Microscopic botanical analyses revealed *Brassica* sp., *Raphanus* sp., and *Sisymbirium* L. in the spring pollen and mainly *Xantihium* spp., *Amaranthus cruentus* L. in the autumn pollen. The protein concentration was 421 µg/mg in the spring mixture and 425 µg/mg in the autumn mixture. Although the total protein content was comparable, differences were observed in the proportions of amino acids. Spring plants contained more amino acids, such as tryptophan, valine, isoleucine, serine, asparagine and glutamine; while autumn plants were poorer in proline and hydroxyproline [41]. The individual composition of pollen of the same species collected from different geographical locations may differ. The total protein content of *Brassica napus* pollen among the tested samples ranged from 18.9% (Saudi Arabia) to 27.3% (China) [26]. The authors found a similar trend in other pollens. This is due to the botanical and genetic origin, soil type, climatic conditions and the activities of beekeepers.

The profile and content of pollen amino acids differs between plant species [42]. De Groot’s [43] division into ‘essential’ and ‘non-essential’ amino acids is justified by presence of arginine, histidine, lysine, phenylalanine, tryptophan, leucine, isoleucine, threonine and valine; and some non-essential amino acids, e.g., alanine, arginine, asparagine, cysteine, glycinma, proline, tyrosine. Our review of the amino acid composition of pollen from selected plants shows that a significant number of plants produce pollen rich in leucine, glutamine, lysine and aspartic acid (Table 1). According to Somerville [44], pollen grains often lack isoleucine and valine. Pollen rich in isoleucine is provided by *Eucalyptus L‘Héritier* de *Brutelle* (Myrtaceae) [22]. Proline and glutamic acids are the dominant exogenous amino acids found in most pollen [45]. The high proline content can also be a marker for assessing the freshness of pollen clusters. If the ratio of proline to all amino acids is lower than 0.65, it is assumed that the pollen samples are fresh and have undergone proper drying treatment [46]. In total, 19 amino acids have been identified in rapeseed, pear and apricot pollen. The contents of valine and leucine in rapeseed and apricot are higher than in pear pollen. Statistically significantly higher phenylalanine concentrations are observed in pear pollen. Moreover, rapeseed pollen has the highest level of tryptophan. It turns out that plants such as *Trifolium pratense*, *Trifolium repens* and plants from the Rosaceae family—that are eagerly visited by honey bee workers—are deficient in one or several amino acids that are necessary for bees [42]. These deficiencies should be supplemented by the insects from other pollen sources and stored in the form of bee bread. Some fluctuations in the amino acid composition are observed in bee bread versus pollen. Bee bread has a higher nutritional value, mainly due to the greater bioavailability of amino acids caused by the action of lactic acid bacteria supplied by the honey bee. This ensures some of the amino acids from proteins are released [46]. Leucine and threonine have 60% higher levels in bee bread [47]. However, bee bread contains significantly lower concentrations of asparagine, proline and aspartic acid compared to pollen grains [26]. For example, the content of essential amino acids (EAA) in corn, eucalyptus and clover pollen is 61.8, 73.0 and 83.5 mg/g, respectively [48].

The nutritional value of pollen also depends on the content of saturated and unsaturated fatty acids. The total lipid content in pollen is 3–20% of its dry weight [4]. The content of individual fatty acids varies depending on the plant species. The most common ones are palmitic acid, linoleic acid and alpha-linolenic acid. In addition to their nutritional properties, decanoic, dodecanoic and myristic acids have antimicrobial properties [2]. Bees also have a nutritional need for sterols, including *β*-sitosterol and 24-methylene-cholesterol—which determine the synthesis of ecdysteroids—inducing the transformation of larvae and the maturation of female ovaries [1,49]. The above-mentioned sterols are not synthesized by flower-visiting insects, so they must be supplied in a pollen diet. Pollen and nectar are also rich in secondary metabolites produced by plants, such as amygdalin [50], quercitin or falvonol. Amygdalin is a cyanogenic glycoside (a plant secondary metabolite) commonly found in the pollen produced by apple trees, cherry trees, etc. [51,52]. Honey bee workers, while pollinating almonds, come into contact with plant tissues that contain high concentrations of amygdalin in their vacuoles. During digestion, amygdalin is broken down into prunasin and glucose. Prunasin is then further broken down into benzaldehyde and hydrogen cyanide. Hydrogen cyanide is toxic to animals, including bees. Nonetheless, forager bees willingly collect almond pollen. On the other hand, amygdalin reduces the titers of some viruses and other microorganisms [51].

In addition to proteins and amino acids, pollen contains small amounts of carbohydrates, lipids, phenolic compounds and fiber [53]. In addition, an important aspect is the stoichiometry of individual elements, especially carbon, nitrogen, sulfur, potassium, sodium, calcium, magnesium, iron, zinc, manganese and copper. Macronutrients and micronutrients play an important role in the functioning of the bee organism as precursors of enzymes and hormones in all metabolic processes [54,55]. Just like humans, insects lack the ability to synthesize most vitamins, so they must ingest them along with pollen. Bee pollen contains about 0.02–0.7% vitamins in relation to its total content [55]. Due to the deficiency of certain vitamins in some pollens [56], supplementation with artificial vitamins administered with sugar syrup or sugar paste is necessary. Water-soluble vitamins, such as those from the B group (thiamine, riboflavin, niacin, folic acid, etc.), dominate in bee pollen. Additionally, bee pollen contains fat-soluble vitamins A, D, E, and K [55]. A plant that produces pollen with a balanced content of the above-mentioned elements is clover (*Trifolium* sp.), while sunflower pollen (*Helianthus* sp.) is characterized by extremely low values of micro- and macroelements [57]. Filipiak and Filipiak [58] showed, in an experiment with solitary bees, that limiting selected elements inhibits the development of larvae and the formation of cocoons in mason bees.

## 3. Recognition of the Nutritional Value of Pollen by Worker Bees

The demand for pollen depends on internal factors such as the number of larvae present in the comb cells, the amount of stored bee bread and the genotype of the bee colony; as well as external (environmental) factors; for example, the seasonal availability of resources, time of day, relative humidity, rainfall wind speed, etc. [10,59,60]. It turns out that the pollen preferences of worker bees depend on the interaction of nutrients in relation to their proportions, rather than being related to a single nutrient. Assessment of pollen quality and nutritional value by workers and nursing bees seems to be difficult because the cytoplasm of pollen grains is surrounded by a thick cell wall [61]. According to research by Beekemna et al. [62], foraging bees are unable to distinguish protein content. This fact is also confirmed by Roulston et al. [63], and by Pernal and Curie [64], who suggest that bees do not prefer diets with higher protein content. In turn, Fewell and Winson [65] found that worker bees are more likely to visit pollen that is rich in nitrogen, which correlates with high protein content. As opposed to the honey bee, research on bumblebees has shown that these insects choose plants with a higher protein content [66]. It is also scientifically confirmed that honey bees collect pollen containing protein and lipids in a ratio of 1:1 to 2:1. For comparison, this ratio in bumblebees is as high as 10:1 [67]. The issue related to the dietary preferences of the honey bee requires further investigation. The diversity of food sources is associated with the possibility of choosing pollen and/or nectar. Human activities related to monocultures (mass flowering of rapeseed) limit the choice of food resources available to honey bee workers [68,69].

## 4. Physiology of Protein Digestion

**Figure 3:**
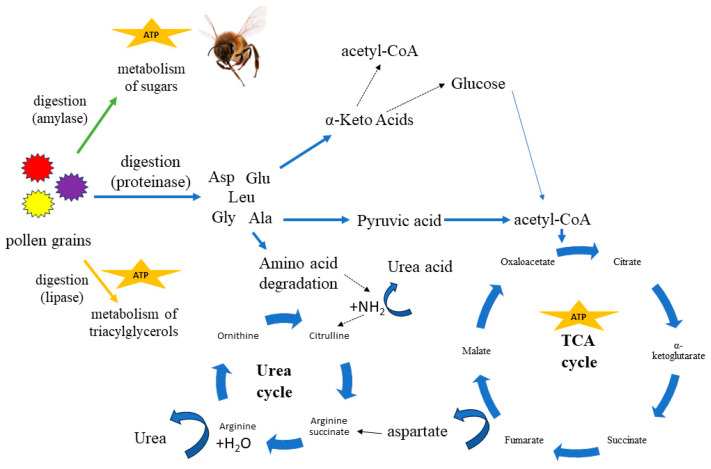
Pollen digestion: amino acid pathway [6,72,73,74,75,76,77,78,79].

A pollen grain is composed of an outer exine layer and an inner layer, where the cytoplasm and cell nucleus are located. Some lipids and amino acids cover the outer pollen membrane. In order to get inside the pollen grain, the cell wall must be digested [46]. Access to the protein stored in the pollen grain depends on the degree of cell wall digestion. Honey bees use osmotic shock to disrupt the pollen wall, digestive enzymes, or mechanical grinding through the mouthpiece [20,63,70]. The average time that pollen stays in the digestive tract of a worker bee ranges from 3 to 24 h. The efficiency of the digestion process is estimated at 75% and is measured by the ratio of digested (empty) grains to intact grains in the feces. Proteins of other origins (e.g., soy) found in artificial diets are digested by bees at a digestive efficiency of 25% [2]. Proteins are digested into amino acids which, like building blocks, form long strings made of tens or even thousands of amino acids. Amino acids can be divided according to the presence of different functional groups, e.g., carboxyl, hydroxyl, or the presence of a ring. Honey bees are invertebrates and share the same protein, lipid and carbohydrate metabolism pathways as mammals. Amino acid metabolic pathways in bees are complicated due to the lack of typical organs such as the vertebrate liver. As in mammals, amino acids can be deaminated or converted into pyruvate and then incorporated into the Krebs cycle (TCA cycle) for the energy to be produced and stored in ATP; e.g., for flight (Figure 3). Most amino acids are transformed into pyruvic acid and then glucose is synthesized. Therefore, some amino acids belong to the group of glucogenic amino acids. The second group are ketogenic amino acids such as phenylalanine, isoleucine, leucine, lysine, tryptophan and tyrosine, which are broken down into acetyl-CoA. From an energy point of view, burning one molecule of pyruvate is more energetic than burning one molecule of acetyl-CoA. The intermediates incorporated into the Krebs cycle come out in the form of malate. The malate will be converted into pyruvate in the mitochondrion and NADPH_2_, and ATP will be produced. A simpler way is to directly incorporate amino acids—e.g., valine and isoleucine—into the Krebs cycle and ultimately produce energy. The final stage of amino acid digestion is the urea cycle. In the urea cycle, ammonia is converted to urea. The enzyme xanthine oxidoreductase converts urea into uric acid. Uric acid is stored in cells in the vacuoles of the fat body [6,71]. Finally, it is excreted with feces.

## 5. The Key Role of Protein and Amino Acids in the Metabolism of *Apis mellifera* Workers

The site of protein synthesis in bees is mainly the fat body, and hemolymph distributes proteins throughout the tissues. Hemocytes are found in the hemolymph. Hemocytes are essential during the body’s response to pathogens in cellular immunity. The number of hemocytes is higher in bees fed a protein-free diet. Pollen diets (*Salix* sp., *Acer* sp., *Cistus* sp.) with high percentages of proteins induce enhanced glucose oxidase (GOX) activities [80]. Consumption of a protein-rich pollen diet leads to an increase in the total protein concentration in seven-day-old worker bee hemolymph relative to worker bees fed sucrose syrup [81]. In addition, the total protein concentrations in the hemolymph of bees fed fermented pollen are about 39 µg/µL after seven days, and are higher than in bees fed fresh pollen (about 32 µg/µL) [81]. Dietary proteins play direct and indirect roles in the insect immune system. The crude protein contained in the bee’s pollen diet determines the synthesis of the isoform of the antimicrobial peptide apidaecin 1. Bees fed with *Castanea* spp. pollen showed the highest increase in the apidaecin 1 isoform compared to pollen from *Helianthus* spp., *Sinapis* spp. and *Asparagus* spp. [82]. Bee pollen also contains short peptides of 3–20 amino acids, which have strong anti-inflammatory properties and free radical scavenging effects, especially against reactive forms of nitrogen [83].

Apart from proteins, smaller fractions, such as amino acids, also play a role in the bodies of pollinating insects. The amino acids leucine, lysine, phenylalanine, valine, methionine, threonine, histidine, isoleucine and tryptophan are not synthesized by bees (exogenous amino acids); therefore, they must be supplied with pollen [1]. Exogenous amino acids have a higher energy value than endogenous amino acids. The largest amounts of amino acids required for the proper development of honey bee workers include: leucine, isoleucine, valine, lysine and threonine [43,84].

Amino acids perform various functions in bees (Table 2). In addition to their functions as building blocks of proteins and polypeptides, they play direct and indirect roles as neurotransmitters. In addition, they are precursors of digestive enzymes, neurohormones and neuropeptides [85]. Amino acids contained in bee pollen consumed in the first five days of life increase the concentration of free amino acids found in the brain tissue of worker bees. The results suggest that access to pollen in early adulthood influences the development of the nervous system [85].

In many insects, especially honey bees, proline is an important amino acid. Proline is synthesized in fat body cells from acetyl-CoA and alanine [86]. The fat body is washed by hemolymph; so amino acids, including proline, enter the hemolymph. The dominant amino acid in the hemolymph of honey bee workers is proline [87]. The average proline concentration in emergence workers is 20 mM. The value of proline fluctuates throughout the life of the worker, but decreases towards the end of life. Amino acids such as methionine, alanine and phenylalanine did not show statistically significant differences [74]. The complete lack of access to pollen in the diet changed the amino acid profile in the nurse’s brain, and this may translate into disorders related to the working behavior of nursing bees [85]. Additionally, an important amino acid in the honey bee’s diet is isoleucine. Honey bees need 4% of isoleucine from pollen. However, isoleucine is a limiting factor and less access to this amino acid will result in a lack of protein bioavailability [88]. This result is consistent with the general statement that pollen rich in essential amino acids is more nutritious than other amino acids. Tryptophan plays an important role in the development, olfactory learning, and memory abilities of honey bee workers [76]. In addition, tryptophan can stimulate the development of the pharyngeal gland, which translates into greater production of royal jelly in nurses [76].

**Table 2:** The role of amino acids in the metabolism of honey bees.

Amino Acids	Role in the Honey Bee Metabolism	Literature
Tryptophan	A precursor of serotonin, a neuromodulator and a hormone whose level in the brain increases with age	[85]
Methionine	The major substitute and active methyl donor for DNA methylation, which is an epigenetic driver of caste differentiation	[89]
Arginine	A substrate used by the enzyme nitric oxide synthase to produce NO, participates in the immune response during injury	[38]
Leucine	Affects many TOR signaling pathways and genes; in insects, as in other animals, it may be associated with the activity of many enzymes such as alanine aminotransferase (ALT), aspartate aminotransferase (AST) and creatine kinase (CK)	[90]
Phenylalanine	Has a strong phagostimulatory effect	[91]
Tyrosine	Participates in in the formation of sclerotin, the matrix in which chitin fibers are embedded	[91]
Histidine	A precursor to histamine	[92]
Cysteine	A limited resource in most insects and is necessary for the production of glutathione; an antioxidant that neutralizes the oxygen forms produced as a result of the reaction and supports immune functions	[93]
Proline	Takes part in physiological changes in temperature, preventing overcooling; proline increases cold tolerance; participates in energy metabolism during flight (energy boost for flight); increases the survival rate and weight of brood larvae	[87,94,95]
Glutamic acid	An important neurotransmitter regulating the processes of learning and memory	[95,96]
Lysine	This amino acid is directly involved in the synthesis of nitric oxide, a known neurotransmitter affecting memory	[85]

## 6. The Phenomenon of Hunger and Low Protein Diversity in a Bee Colony Conditioned by a Mono Diet and Invasive Plants

Stressful situations caused by periods of starvation for bee colonies are very dangerous. The first symptom associated with starvation is inhibited brood rearing. Then the queen bee stops laying unfertilized eggs, and eggs and young larvae may be eaten by adult worker bees to obtain protein [97]. Additionally, during starvation, disturbed worker bee behaviors such as ejecting larvae outside the hive or disrupted hygiene mechanisms are observed, leading to the development of diseases [56,98]. Starvation is conditioned by either a complete lack of access to food or an unbalanced access to nutrients caused by a mono diet. The problem of the declining diversity of these fodder plants, including: monocrop plants [99], causes nutritional stress [100]. An important effect on pollinators is exerted by vegetation growing on field margins, fallow lands, roadsides and railway embankments [99]. Such vegetation provides extended development and diversity, depending on the environment. Weeds in field margins also diversify pollen production. Unfortunately, most farmland is acres long, providing insects with an invasive monopoly on the agricultural landscape. An example of a mass-grown crop is rapeseed [101]. This plant provides huge amounts of pollen and nectar, which makes it a very attractive food source for honey bees [102]. The percentage of protein in rapeseed pollen is approximately 27% [78,103]. Unfortunately, the crop is often treated with pesticides, which poses a secondary threat to the honey bee. *Phacelium* and *buckwheat* are also widely cultivated on a large scale. Phacelia pollen (*Phacelia tanacetifolia* Benh.) contains 27.44% soluble, and other organic compounds such as beta-carotene [27]. In contrast, buckwheat pollen contains approximately 11.4% of protein [44]. Our findings show that buckwheat pollen has the lowest protein content in comparison with other pollen plants mentioned in this study. Large-scale farms are a common phenomenon in Europe and in China. In this case, pollinating insects have access to large amounts of pollen not only from rapeseed, but also from pear and apricot trees. The protein content in monocultures may be low and the amino acid composition not very diverse. Moreover, the periods of rapeseed, apricot and pear flowering overlap and bees are observed to have flower preferences. According to Chang et al. [76], more frequent visits to rapeseed flowers, as opposed to pear flowers, result from the higher sugar content in the rapeseed nectar. In a laboratory experiment, bees fed a mono diet showed a significant preference for apricot pollen over pear pollen. This fact may indicate an amino acid preference. Color and olfactory preferences cannot be ruled out either.

Nutrient deficiency and the natural phenomenon of a mono diet may be caused by pollen from invasive plants, due to honey bees visiting both native plants and invasive plants. Additionally, it is said that as invasive plant species increase, the number of native flower-visiting insects decreases. Nevertheless, the honey bee successfully pollinates some invasive plants. According to a study of pollinator visits conducted by Salisbury et al. [104], higher numbers of pollinators are found on native and near-native plant species than on exotic species. Pollinating insects are reluctant to visit newly introduced plant species, unlike flowers that occur naturally in a given climate zone [51,105]. The reluctance may be due to the low sugar content in the nectar or the poor chemical composition of the pollen, especially low amino acid levels. Pollen from invasive plants, such as *Buddleia davidii* Franch and *Impatiens glandulifera* Royle, contained lower concentrations of amino acids than native species [106]. Goldenrod provides pollen and nectar for honey bees in late summer and early autumn. The total protein content in the pollen produced by *Solidago* spp. is over 20% [28]. The expansion of the invasive Solidago sp. species determines the natural creation of the mono diet [99]. According to Denisow [107], several plant species in the Polish climatic zone that bloom in late summer—e.g., *Echium vulgare* L., *Medicago sativa* L. and *Melilotus officinalis* L.—produce pollen with a higher total protein content. Nevertheless, goldenrod is considered a major source of protein due to its abundant flowering. Goldenrod (*Solidago canadensis* L.) globally creates high competition for native fauna. Due to the rapid expansion of its range through seed dispersal, vegetative reproduction, rapid growth dynamics, and the production of allelopathic compounds that inhibit the growth of neighboring plants, goldenrod is categorized as an invasive species [108]. Pollen and nectar secreted by goldenrod are an important late autumn food for pollinators in North America and Europe, including Poland, stored in the form of bee bread and honey in bee combs [109]. Goldenrod honey is being promoted for its unique properties, driving the demand for it [110]. Discussions among beekeepers on online forums about goldenrod are divided. We are unable to answer the question: How does this plant affect the wintering of bees, and especially the immune processes in bees? Therefore, there is a need for research on a goldenrod pollen-based diet.

The global problem of declining quality and shortage of pollen determines the human search for alternative methods of providing protein to honey bee workers. There are a number of tested supplements based on soy protein, skimmed milk powder, egg yolk powder, casein or fish meal, the ingredients of which were tested on *Apis mellifera* L. colonies [9,111,112]. They stimulate the development of the brood, determine the development of the hypopharyngeal glands (HPG), and all this translates into honey production in the colonies. There are also yeast–gluten mixtures available on the market with the addition of vitamins, amino acids, pollen, etc. It has been reported that the mixtures are perfect for periods of malnutrition and have a positive effect on colony parameters [9,113,114]. Bees fed with pollen, fishmeal and sugar were compared. Sugar had no effect, fishmeal worked satisfactorily, and pollen was the best in terms of bee colony development rate [9].

## 7. Conclusions and Further Research Directions

Bee pollen is one of the best sources of digestible protein for worker bees. Metabolites formed as a result of protein digestion play an important role in their physiology, especially energy production. Unavailability of one of the amino acids may be a factor that limits individual development. More research is needed on the protein content of worker pollen diets in relation to effects on biochemical parameters such as protein concentration, amino acid and fat content in the hemolymph and fat body. The cited parameters correlate with over-wintering, royal jelly production and honey bee immunity. In addition to the protein contained in pollen, other parameters should be considered, such as elemental composition and sterols etc. The influence of pollen produced by monoculture plants, such as rapeseed, and invasive plants, such as goldenrod, *Solidago* spp., on proper overwintering and resistance, cannot be overlooked.

## Figures and Tables

**Table 1:** Comparison of total protein content and amino acid composition in pollen (based on Google Scholar and Scopus).

Taxon	Total Protein Content [%]	Dominant Amino Acid Composition	Literature
*Brassica napus*	from 22 to 27	Aspartic acid, Glutamic acid, Lysine, Leucine	[22,23,24,25,26]
*Phacelia tanacetifolia*	27.44	Glutamic acid, Proline, Aspartic acid, Leucine, Lysine, Valine	[27]
*Solidago gigantea; Solidago canadensis*	>20	No literature data available	[28]
*Fagopyrum*	11.4	Glutamic acid, Proline, Aspartic acid, Leucine, Tryptophan, Lysine, Valine, Alanine, Arginine	[22,25]
*Medicago sativa*	20.23	Valine, Leucine, Izoleucine Phenylalanine, Proline	[24]
*Phoenix dactylifera*	19.77	Methionine, Histidine, Glycine, Alanine	[24]
*Vicia faba*	from 22 to 24	Proline, Aspartic acid, Glutamic acid, Arginine, Leucine, Tryptophan	[29,30]
*Helianthus annus*	15.19	Leucine, Valine, Lysine, Histidine, Aspartic acid, Arginine, Tryptophan, Glutamic acid	[24,25,29]
*Zea mays*	14.9	Proline, Aspartic acid, Lysine, Alanine, Arginine, Tryptophan	[22,26]
*Eucalyptus bridgesiana:*	23.1	Proline, Glutamic acid, Aspartic acid, Leucine	[22]
*Echium plantagineum*	37.4	Aspartic acid, Glutamic acid, Leucine, Lysine	[22]
*Salix discolour*	21.9	Glutamic acid, Aspartic acid, Leucine, Lysine	[22]
*Castanea sativa*	21.6	Proline, Aspartic acid, Glutamic acid	[31,32]
*Rubus* sp.	22	Leucine, Lysine, Valine, Phenylalanine, Threonine, Izoleucine	[31,33]
*Sinapis*	No literature data available	Aspartic acid, Glutamic acid, Proline, Lysine	[34]
*Acacia* sp.	21.8	Aspartic acid, Glutamic, Glycine	[35]
*Calluna vulgaris*	17	Glutamic, Aspartic acid, Glycine	[36]

## Data Availability

No new data were created.
